# Parasitism to mutualism continuum for Joshua trees inoculated with different communities of arbuscular mycorrhizal fungi from a desert elevation gradient

**DOI:** 10.1371/journal.pone.0256068

**Published:** 2021-08-27

**Authors:** Jennifer T. Harrower, Gregory S. Gilbert

**Affiliations:** Department of Environmental Studies, University of California, Santa Cruz, Santa Cruz, California, United States of America; Institute for Sustainable Plant Protection, C.N.R., ITALY

## Abstract

Most desert plants form symbiotic relationships with arbuscular mycorrhizal fungi (AMF), yet fungal identity and impacts on host plants remain largely unknown. Despite widespread recognition of the importance of AMF relationships for plant functioning, we do not know how fungal community structure changes across a desert climate gradient, nor the impacts of different fungal communities on host plant species. Because climate change can shape the distribution of species through effects on species interactions, knowing how the ranges of symbiotic partners are geographically structured and the outcomes of those species interactions informs theory and improves management recommendations. Here we used high throughput sequencing to examine the AMF community of Joshua trees along a climate gradient in Joshua Tree National Park. We then used a range of performance measures and abiotic factors to evaluate how different AMF communities may affect Joshua tree fitness. We found that fungal communities change with elevation resulting in a spectrum of interaction outcomes from mutualism to parasitism that changed with the developmental stage of the plant. Nutrient accumulation and the mycorrhizal growth response of Joshua tree seedlings inoculated with fungi from the lowest (warmest) elevations was first negative, but after 9 months had surpassed that of plants with other fungal treatments. This indicates that low elevation fungi are costly for the plant to initiate symbiosis, yet confer benefits over time. The strong relationship between AMF community and plant growth suggests that variation in AMF community may have long term consequences for plant populations along an elevation gradient.

## Introduction

Symbioses between plants and mycorrhizal fungi are ubiquitous and diverse. This ancient interaction evolved over 450 million years ago and is credited with assisting the early colonization of land by plants [1–4]. In exchange for carbon, the fungi provide greater access to soil nutrients and help plants resist disease, salinity, and drought, thereby playing a key role in plant fitness, productivity, and community composition [5–7]. However, these fungal symbionts are not always beneficial to the plant host; the fungi can sometimes function as parasites, reducing performance of their plant partners [8–10], depending on the species involved [11], the environmental conditions where the interaction occurs [12, 13] and developmental and phenological factors [14, 15]. The outcomes of the interactions are mutualistic when net benefits are greater than net costs for both partners, commensal when one partner benefits but the other receives neither benefit nor harm, and parasitic for either partner when costs exceed the benefits received [8, 15, 16].

The distribution of arbuscular mycorrhizal fungal (AMF) is regulated by environmental parameters such as soil nutrients, texture [17] and pH [18], disturbance [5] as well as availability of suitable plant hosts [19, 20]. As plant performance [21] and symbiotic outcomes [9, 10] are linked to fungal community composition, understanding how local abiotic and biotic factors affect fungal distribution will inform management decisions for specific ecosystems and targeted species [21].

Most plants form symbiotic associations with a diverse assemblage of mycorrhizal fungi, and while progress has been made to elucidate the ecological factors that shape fungal distribution and abundance [22], the mycorrhizal outcomes of different partners interacting across different locations remains poorly understood. Determining the numbers, identities, and distributions of the fungi involved is the first step towards understanding their ecosystem role and host impacts, because for many plants, changes in the AMF community result in changes to a plant’s mycorrhizal growth response (MGR) and ability to accumulate nutrients [19, 23]. For example, inoculating the host plant *Medicago truncatula* with three different AMF species resulted in different outcomes depending on the AMF species [14]. Even different isolates of the same fungal species can differ greatly in their ability to reward the host plant, with great variability in functional response of a single species of AMF depending on biotic and abiotic factors [24]. In another study, a single pair of symbiotic partners (*Petunia hybrida* and *Rhizophagus irregularis*) generated the entire range of mutualistic to parasitic outcomes depending on the nutritional conditions under which the interaction occurs [13]. A series of experiments to understand mycorrhizal function in grassland plants inoculated with native and foreign AMF also resulted in the entire spectrum of symbiotic outcomes [25].

AMF community patterns have been studied across changing environmental and elevational gradients [26–30], yet knowledge about fungal community composition along elevation gradients in desert environments is limited [30, 31]. Elevation gradients can create strong ecological gradients over short geographic distances through corresponding variation in weather patterns, temperature, soil moisture, nutrients, and species distributions [29, 30, 32]. Mycorrhizal symbiosis could be particularly important at the extremes of environmental gradients, helping to ameliorate the effects of stressful conditions on plants [31].

The contingent nature of plant-AMF symbioses makes difficult predicting how climate change will affect the outcomes of mycorrhizal interactions. The benefit to a plant host species depends on the assemblage of fungi available in the location as well as the abiotic conditions that shape the interaction, both of which vary spatially. The combination of molecular observational studies to document current patterns of fungal species distributions across environmental gradients and controlled experiments to measure impacts of mycorrhizal symbioses will help us to predict symbiotic outcomes under future climate scenarios.

To better understand how the role of climate, soil conditions, and fungal species influence symbiotic outcomes on a culturally significant plant in a desert environment, we focused on the mycorrhizal community of the Joshua tree (*Yucca brevifolia)* along an elevation gradient in Joshua Tree National Park (JTNP). Joshua trees are icons of the Mojave Desert, and are threatened by the rapidly changing climate [33]. The temperature in the Mojave has been steadily increasing, and models predict the climate in JTNP will soon be outside of the range of tolerance of Joshua trees, leading to the potential extirpation of Joshua trees from their namesake park within the next century [34–36]. As the Southernmost portion of the Joshua tree range occurs within JTNP, there is a unique opportunity to study these mycorrhizal interactions across a climate stress gradient within the park, and how changes in abiotic conditions that influence mycorrhizal symbiosis outcomes may affect current and future populations of a threatened plant species. For many plants, changes in AMF community result in changes to a plant’s mycorrhizal growth response (MGR) and ability to accumulate nutrients [37–39], but we do not know what role these fungi play for Joshua trees.

**Fig 1:**
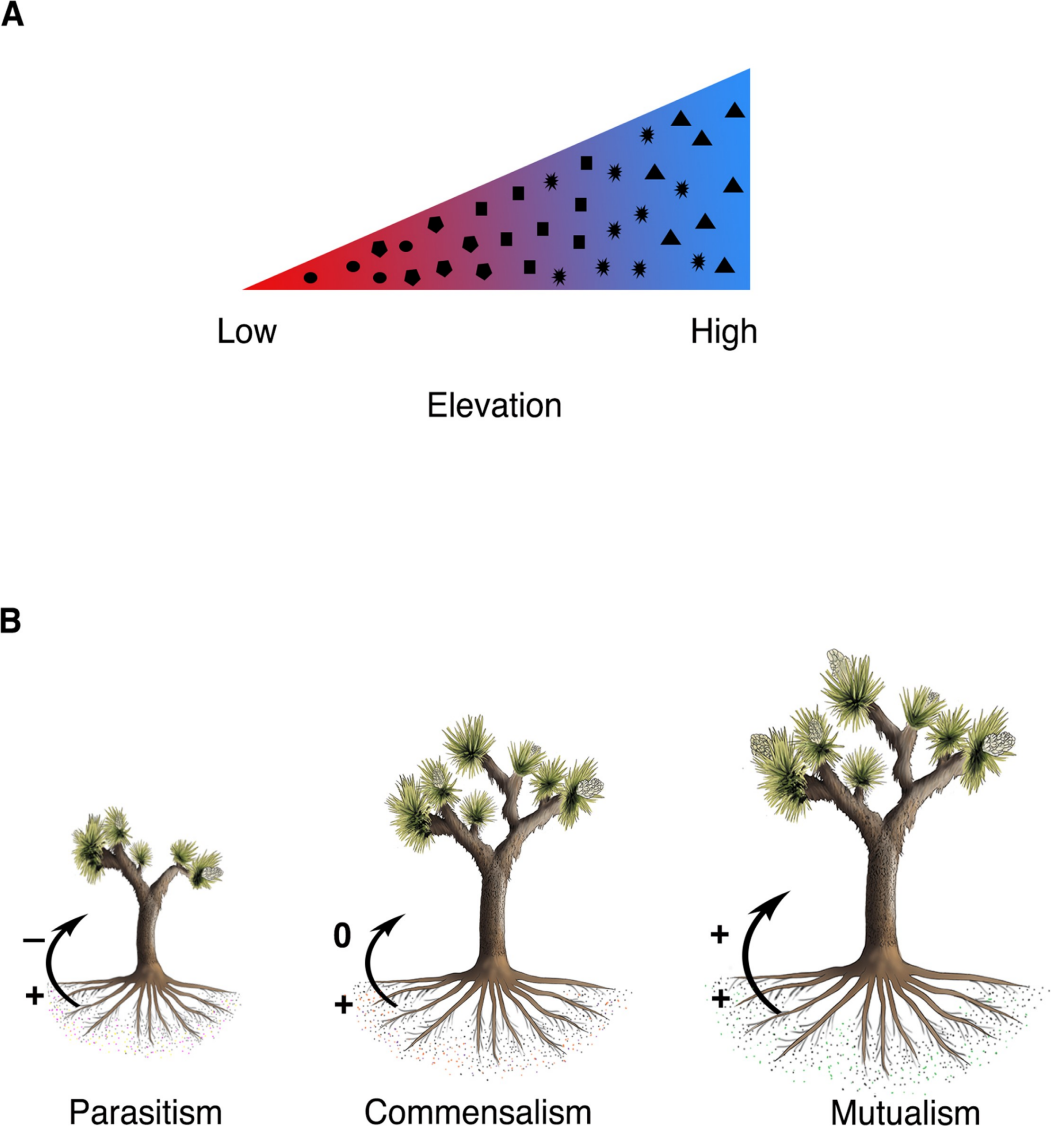
Abiotic and biotic effects on plant-AMF mutualistic outcomes when inoculated with fungi from an elevation gradient. A) Abiotic effects: AMF community composition in Joshua tree roots changes along an elevation gradient. Elevation gradient in the figure corresponds to a changing climate gradient with warmer temperatures at the lowest elevations and the coolest temperatures at high elevations. Changes in fungal communities are represented by the assorted changing black shapes. B) Biotic effects: Variation in the AMF community correspond to different symbiotic outcomes for Joshua trees. Outcomes can range from positive to negative with the arrow demonstrating fungal parasitism (+, -) of the tree, commensalism (+, 0) with the fungus benefitting but not damaging the tree, or mutualism (+, +) with both the tree and the fungi benefitting.

This study is guided by a conceptual model informed by our understanding of how abiotic conditions shape the ecological outcomes of mycorrhizal symbioses. Our model (Fig 1) characterizes the expected drivers and structure of the range of symbiotic outcomes on a parasitism-mutualism spectrum. We use molecular tools to characterize associations between abiotic conditions and the desert AMF community composition and structure (Fig 1A), and then controlled greenhouse experiments to measure the functional impacts of variation in fungi on plant performance (Fig 1B).

In this study, we examined how the AMF community that associates with Joshua trees varies across an elevation gradient in JTNP and how those different fungal partners may impact Joshua tree fitness. Specifically, we ask: 1) Does the AMF community composition in Joshua tree roots change along an elevation gradient? 2) Does variation in the AMF community correspond to different outcomes of the symbiosis for Joshua trees? 3) Do those outcomes change with time as the plants grow?

## Materials and methods

### Study site and sampling methods

The study was conducted across a 1200-m elevation gradient in Joshua Tree National Park (JTNP) (located at 33.8734° N, 115.9010° W) southwestern California, USA, in Spring 2016. JTNP encompasses both the Mojave and Colorado deserts, and varies from hot and dry at low elevations, to seasonal freezing at high elevations [33]. Eleven study sites were selected for analysis, from the low elevation southern Joshua tree range limit, to the high elevation at the northern end of the local distribution. We obtained climate and soil moisture data for sites from six HOBO Pro V2 datalogger weather stations (Onset Computer, Cape Cod, Massachusetts, USA). A description of patterns of weather conditions and soil nutrients along the elevation gradient (S1 Table) has been described previously for these sites [33]. Briefly, average summer temperature declined with increasing elevation (r^2^ = 0.9), ranging from 30.2°C to 19.9°C. Similarly, relative humidity (r^2^ = 0.636) and soil moisture (r^2^ = 0.8) generally increased with elevation. Except for a decline in pH from 8.06 to 6.63 with increasing elevation (r^2^ = 0.827), soil nutrients did not follow any particular elevational trend. Data was collected under permit #JOTR-2117-SCI-0008 approved by vegetation branch chief Michael Vamstead.

To collect and identify AMF communities, we sampled Joshua tree roots and associated soil from 3 randomly chosen individuals at each of the 11 sites. Soil for nutrient and fungal molecular analysis was sampled in triplicate at the base of each tree using a soil core (5 or 10 cm diameter, 15 cm deep), and then pooled for each site 9 cores/site (S1 Fig). Climate, soil moisture, and soil nutrient data from these sites have been presented previously [33] as part of analysis of Joshua tree demographic patterns; we include them again here (S1 Table) as explanatory variables for analysis of variation in AMF communities. To collect fungal inoculum, fine roots and bulk soil were collected from each tree by digging along large roots and collecting 3 samples of root/soil mix from around the tree, thus ensuring that sampled roots belonged to the Joshua tree (2.27 L / tree), 3 trees per site, and then pooled by site (S1 Fig). After collection, samples were placed into individual bags and kept on ice, until they were either transferred to a -20°C freezer where they were stored until the roots were used in DNA extraction, or used for soil nutrient analysis. Soil was dried, ground, and analyzed for total carbon and total nitrogen following the combustion method (AOAC, 1997), pH (in H_2_O), total extractable ammonium and nitrate content by flow injection analyzer method [40, 41], extractable phosphorus using the Olsen method [42], and percentage soil moisture following the gravimetric method [43]. Analyses were performed at the UC Davis Analytical Lab (http://anlab.ucdavis.edu; S1 Table).

### Intraradical AMF colonization

We used root staining to visually determine the extent of root colonization of AMF in root samples taken from each individual Joshua tree at each site. We washed roots free of debris using a 0.7-mm soil sieve, cleared the roots in boiling 10% KOH, neutralized in 5% HCl, and stained in 0.1% trypan blue to visualize colonization by AMF [44]. Stained roots were selected randomly and placed on a slide, and percent root colonization was estimated using the modified line-intercept method [44, 45], scoring the quantity of AM fungal structures (hyphae, vesicles, and arbuscules).

### Plant inoculation, sampling, and nutrient extraction methods

Seeds of *Y*. *brevifolia* were collected and pooled from Joshua tree pods collected across the elevation gradient in JTNP in September 2014 (the lowest and highest elevation sites did not have seed pods–see reference [33] for a discussion on this). The seeds were surface sterilized with 70% ethanol followed by a 0.5% sodium hypochlorite solution diluted in deionized water, and then rinsed with sterile, deionized water and germinated on moist filter paper in complete darkness for 3 days.

To establish the mycorrhizal symbiosis, three germinated seeds were placed in each of 144 Ray Leach UV-stabilized Cone-tainers (164 mL) (Stuewe and Sons., Inc. Oregon, USA) filled with a twice-autoclaved mixture (1 h, 120° C, and then again after 24 h of rest) of sand (70% quartz sand, 0.125–0.25 mm) and greenhouse soil (30% Pro-mix HP). Seedlings were thinned to 1 per pot after 2 weeks. A 1-g band of AMF community inoculum (roots and soil) collected at each of the 11 sites, was added 2 cm below the soil surface, one treatment per pot at the time of planting [46]. Each pot received 1 ml of an AMF-free microbial filtrate wash produced from a mix of all samples and filtered with a 20μm, to correct for possible differences in microbial communities [47]. Non-mycorrhizal controls receiving only the AMF-free filtrate, resulting in a total of 144 experimental units with 6 replicates per treatment. The inoculated seedlings were grown in a randomized complete block design at the UC Santa Cruz Greenhouses, and rotated every 3 weeks to minimize differences from environmental effects in the greenhouse. Soils were watered twice per week and fertilized at 2, 5, and 8 months with a half-strength Hoagland’s solution [48].

Plants were destructively harvested (in triplicate) at 1, 3, 6, and 9 months. Plant biomass was assessed as a proxy for plant fitness. Whole plants (roots and shoots) were oven dried at 55°C for 3 days and then weighed. After weighing, a subsample of roots from each plant was rehydrated, washed, stained with 0.05% Trypan Blue and examined at 40x to confirm the presence of AMF colonization [45].

Dried whole plants were ground with a Wiley Mill to pass through a 40-mesh screen, and then analyzed for total nitrogen following the combustion method coupled with gas chromatography, total phosphorus and potassium utilizing a nitric acid/hydrogen peroxide microwave digestion and determined by Inductively Coupled Plasma Atomic Emission Spectrometry at the UC Davis Analytical Lab (http://anlab.ucdavis.edu).

### Molecular analysis of fungal inoculum

We extracted DNA from 30–80 mg of field sampled and frozen roots for each pooled sample with a PowerSoil-htp soil DNA isolation kit (MO BIO laboratories, Inc., Carlsbad, CA, USA). We made two modifications to the manufacturer’s standard protocol to increase DNA yield; bead plates were shaken at an elevated temperature (60° C) and the final elution was performed twice.

DNA from root samples was sequenced using the amplicon-based Illumina MiSeq platform. Nuclear SSU rRNA amplicons were generated with primers NS31 and AML2 [49] to identify AM fungi. Amplified DNA was purified with the Qiagen QIAquick Gel Extraction kit (Qiagen Gmbh, Hilden, Germany) following the manufacturer’s protocol, and quantified with a Qubit 2.0 Fluorometer (Invitrogen, Grand Island, USA). DNA was processed for sequencing libraries with the Illumina Nextera XT sample preparation kit (Illumina, San Diego, USA) following the manufacturer’s protocol, including tagmentation to fragment and tag DNA, followed by a 12-cycle PCR to add sequencing indices, PCR cleanup, and normalization. Libraries were then sequenced on Illumina MiSeq with 2 x 250 bp paired-end reads at the Research and Testing Laboratory sequencing facility in Lubbock Texas (http://www.medicalbiofilm.org/).

For bioinformatics analysis, we generated multiple sequence alignments with MAFFT (v7.306, [50]) and combined paired-end reads with FLASh (v1.2.10, [51]). The reads were quality-filtered by removing sequences with Nextera adapter contamination from tagmentation, and where average quality was <30. Paired-end reads were trimmed to retain the most variable section of the amplicon, and combined using FLASh with the default parameters (minimum overlap between paired reads = 10bp, maximum mismatch density in overlap = 0.25). We then removed chimeric reads with USEARCH (v7.0.1090, [52]) in reference database mode (MaarjAM database, [53]), clustered sequences with blastclust (BLAST v2.2.26, [54]) and identified sequences with BLAST. The MaarjAM database contains sequences covering the NS31/AML2 amplicon which are classified into virtual taxa (VT) [53]. A virtual taxon is a phylogenetically defined group of closely related SSU rRNA gene sequences with sequence identity equal to or higher than 97%, and anchored to type sequences. From the 11 sites, we had 7 678 300 raw reads in total–with 210 035 to 1 100 345 reads per sample. After adapter removal and quality filtering, 799 748 quality filtered sequences remained. Paired reads were quality filtered and trimmed to the most variable region of the amplicon leaving 210 106 sequences. Taxonomic assignment was given by blasting the representative sequences against the MaarjAM database (https://maarjam.botany.ut.ee), and sequences were assigned to VT when sequence similarity was ≥97%. Reads that did not match against the MaarjAM database were identified with BLAST using the NCBI database with a 97% identity threshold.

### Constructing the phylogenetic tree

We constructed the bootstrap consensus tree using MEGA v7.0, by aligning representative OTU’s with MUSCLE and then assembling those sequences into a neighbor-joining tree. One thousand rapid bootstrap replicates were built and used to apply a Maximum Composite Likelihood model for determining the evolutionary connections among the sequences [55].

### Statistical analysis

We conducted simple linear regressions to evaluate relationships between elevation and soil characteristics. We then used generalized additive models (GAM) to describe the non-linear relationship between elevation and the % colonization of Joshua tree roots by AMF. GAMs are nonparametric extensions of linear models that allow the expected response to vary smoothly with a set of predictor variables [56]. We used the dry plant biomass of plants with or without AMF as a proxy to calculate the mycorrhizal growth response (MGR), and the effect of different AMF treatments on plant fitness [15]. The MGR was calculated as the log response ratio, MGR = log(biomass treatment/biomass control) [57]. Positive values of MGR indicate that the plant biomass increased following inoculation, while negative values indicate a decrease in biomass in response to the fungi.

To examine the relationship between AMF taxa and elevation we carried out a principal component analysis (PCA) of the 11 sites based on the presence of fungal taxa. The PCA of the 11 sites allows visualization of the data and demonstrates how the AMF are distributed among the sites and in relation to each other in a multivariate space. All calculations were performed using the R language for statistical computing with the following library packages: ggplot2, reshape2, plyr, ape, RColorBrewer, mgcv, broom, and tidyverse (The R Development Core Team 2018).

## Results

### Percent AMF colonization

**Fig 2:**
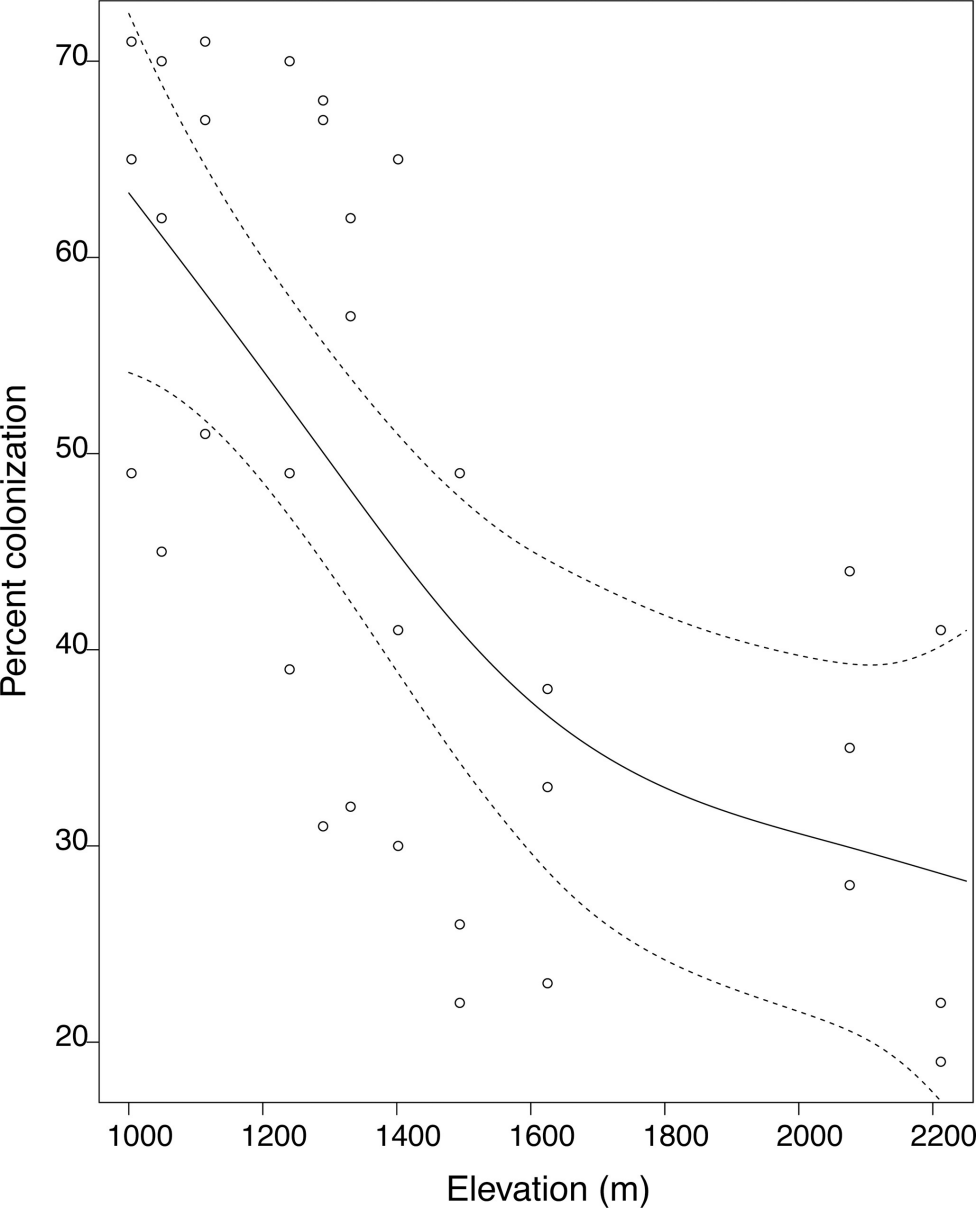
Percent mycorrhizal colonization of Joshua tree roots measured as a function of elevation. Solid line represents the fitted values from a generalized additive model that estimates the form of a relationship between the thirty-three root samples and elevation. Points show values for each of the three samples taken at a site. GAM fit indicates a greater degree of colonization for roots at lower elevations than higher elevations. Dotted lines represent 95% confidence intervals.

The percentage of root length colonized by AMF varied from 19 to 71% with an average of 46.7%, (SD = 17.1). In the generalized additive models, mycorrhizal colonization decreased significantly with increasing elevation (r^2^ = 0.48; p = 2.1x10^-4^) (Fig 2).

### Fungal community composition of inocula

**Fig 3:**
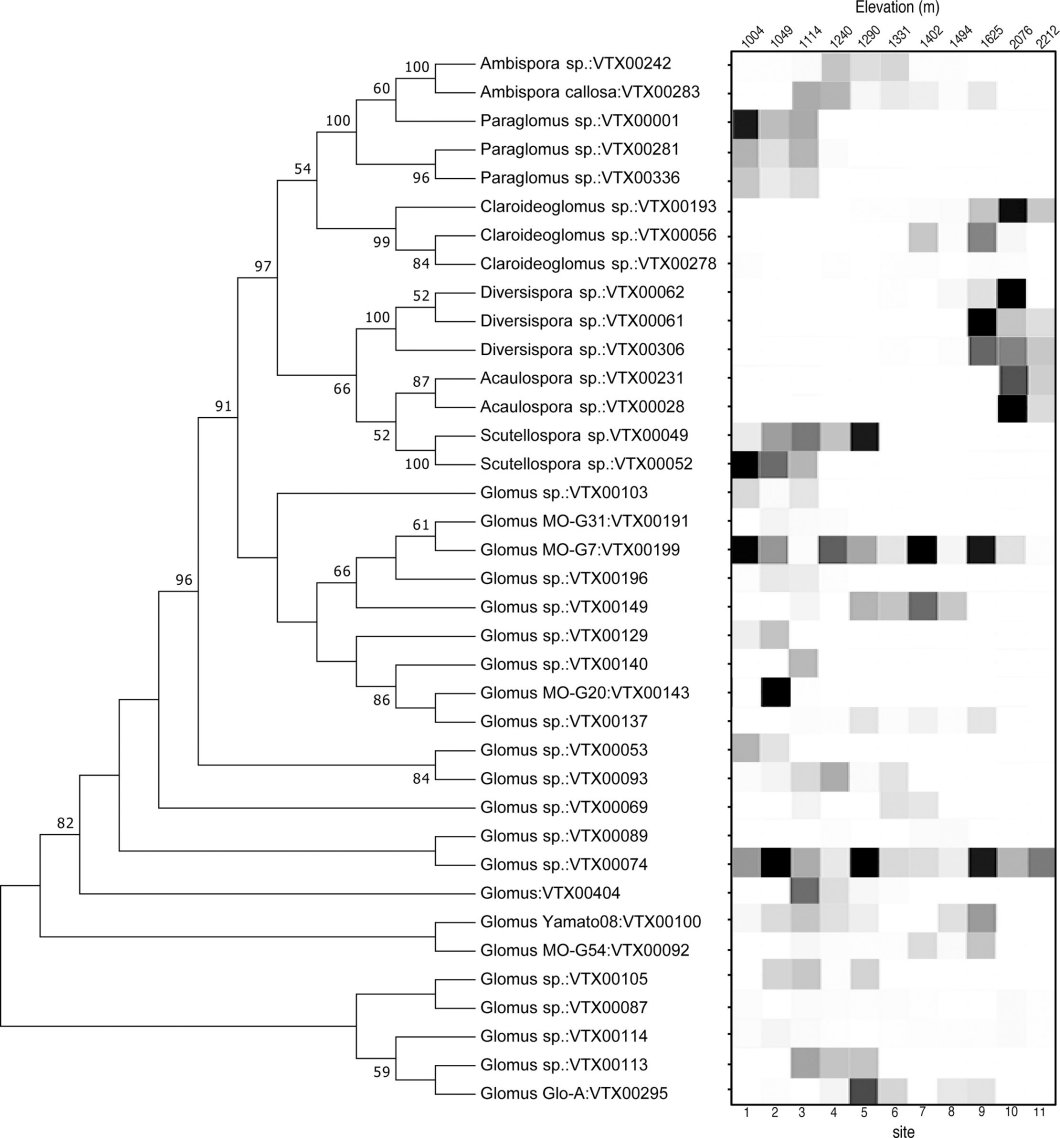
Bootstrap consensus tree and heatmap of the relative abundances of each AMF virtual taxon (VT) across the elevation gradient in Joshua Tree National Park, CA, USA. The tree was built using the Maximum Composite Likelihood method [55] and evolutionary analysis conducted in MEGA7. Node numbers represent Bootstrap values with only those values above 50 displayed. Tips represent AMF species name and VT as they are listed in the MaarjAM database. For the heatmap, rows represent the presence of AMF and the darkness of square indicates the read abundance expressed on a scale from 1–10 of each VT shown on the tree, at each elevation sampled.

**Fig 4:**
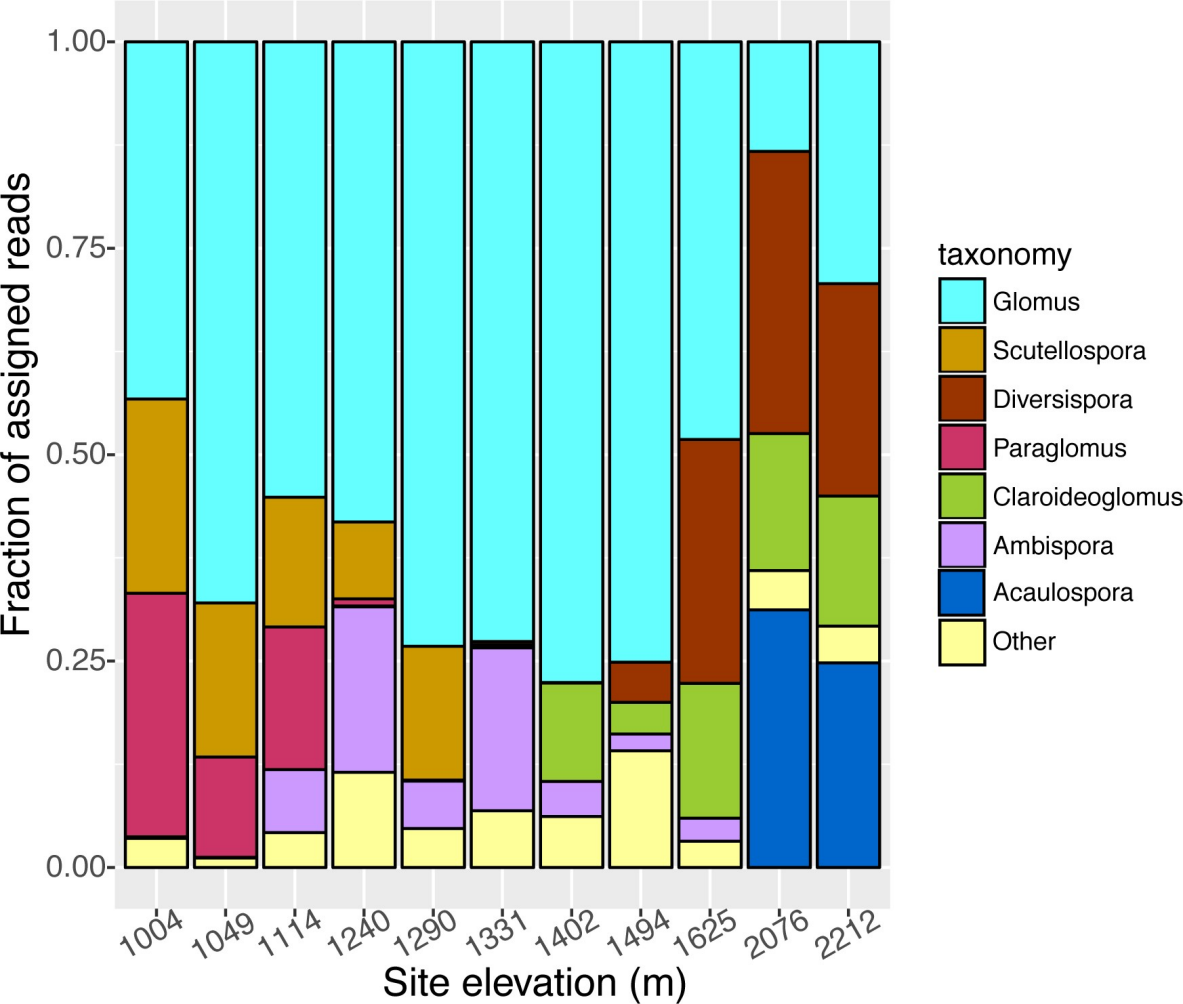
Relative abundances of genera of AMF taxa colonizing Joshua tree roots harvested across an elevation gradient in Joshua Tree National Park. Abundance is estimated as the proportion of assigned sequence reads within each site.

We identified 37 virtual taxa (VT) including sequences from 7 genera in the phylum Glomeromycota. The phylogenetic placement of the different AMF VT’s were determined with a neighbor-joining tree, and shown with the relative abundance for each VT (Fig 3). The most abundant taxa were in the families: Glomeraceae, followed by Gigasporaceae, Diversiporaceae, Paraglomeraceae, Claroidioglomeraceae, Ambisporaceae, and Acaulosporaceae (Fig 4). We detected significant variation in AMF community composition at the genus level across the 11 sites. *Glomus* was the most abundant genus across all samples. *Paraglomus* and *Scutellospora* were only found in the lowest elevation sites (1004–1240 m and 1004–1290 m, respectfully). In contrast, *Ambispora* was distributed across the middle elevations, from 1114 to 1625 m. *Claroideoglomus* was found from mid to high elevations at 1290 to 2212 m, while *Diversispora* was located at the four highest elevation sites from 1494 to 2212. *Acaulospora* was only found at the two highest sites, 2076 m and 2212 m. (Data publicly accessible in NCBI repository SRA submission: PRJNA755700).

**Fig 5:**
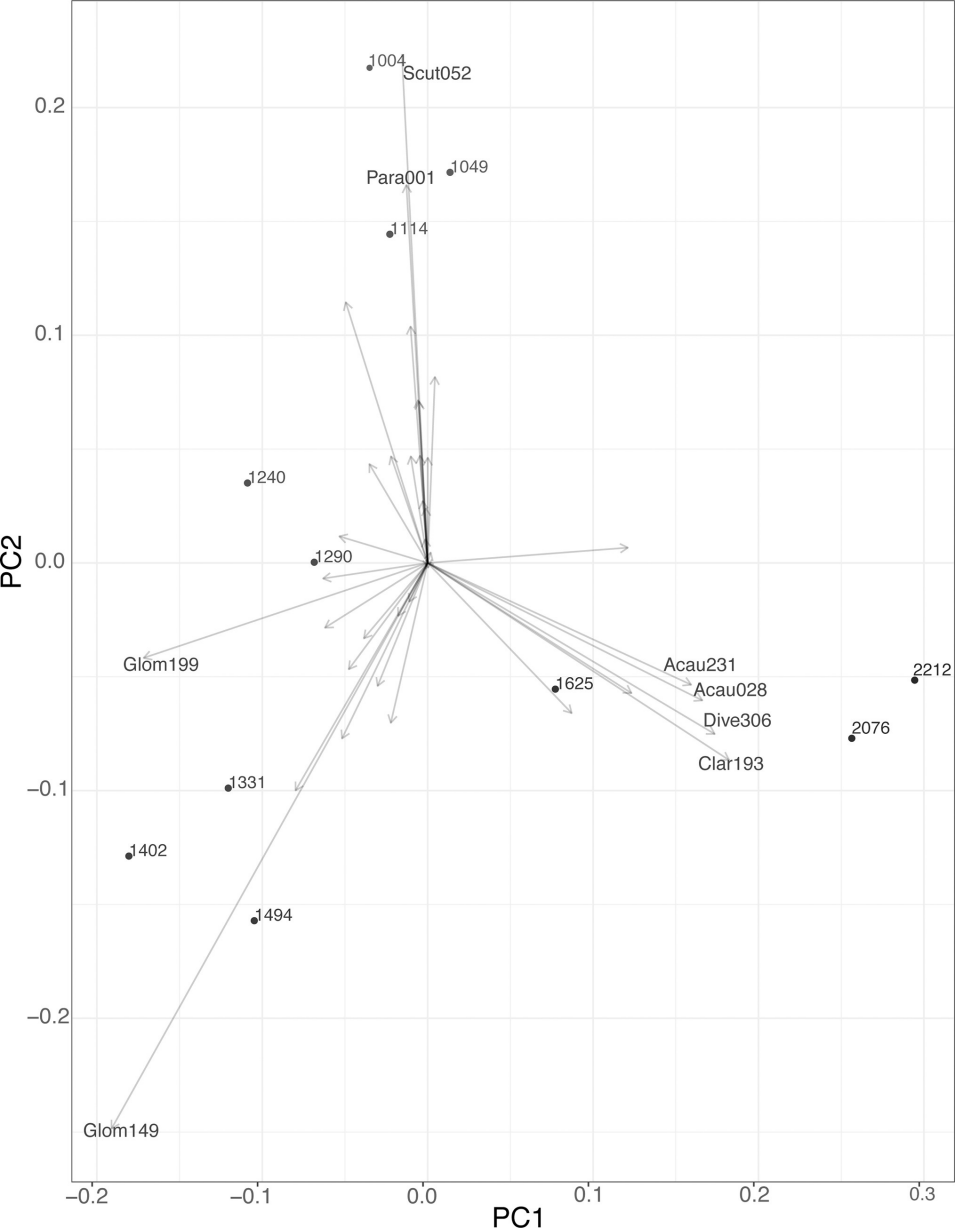
Biplot of first two components (PC1 and PC2) of a principal component analysis of eleven sites, based on the relative abundance of major fungal taxa. Each point is labeled with the elevation in meters of one of the eleven sample sites. The arrows represent the loadings for each AMF taxon across all sites. The 8 taxa with the strongest loadings are labeled using the first 4 letters in the genus name, and the last three numbers in the virtual taxa ID, for example: Scutellospora sp.:VTX00052 = Scut052.

In the principal component analysis, PC1 explained 31% and PC2 explained 21% of the variation in fungal composition across sites (Fig 5). The 11 sites show strong grouping of fungal taxa by elevation, with low (1004–1114 m), mid (1240–1494 m), and high elevation sites (1625–2212 m) clustered together with similar AMF composition (hereafter referred to as low, mid or high elevation fungi). *Scutellospora* and *Paraglomus* dominated at the low elevation sites, *Glomus sp*. and *Ambispora* at the mid elevations, and *Diversispora*, *Claroideoglomus*, and *Acaulospora* at the high elevation. Only taxa with the 8 strongest loadings are shown (Fig 5), as a plot of vector rank as a function of vector length drops off after the first 8 taxa (S1 Fig).

### Plant-mycorrhizal response

**Fig 6:**
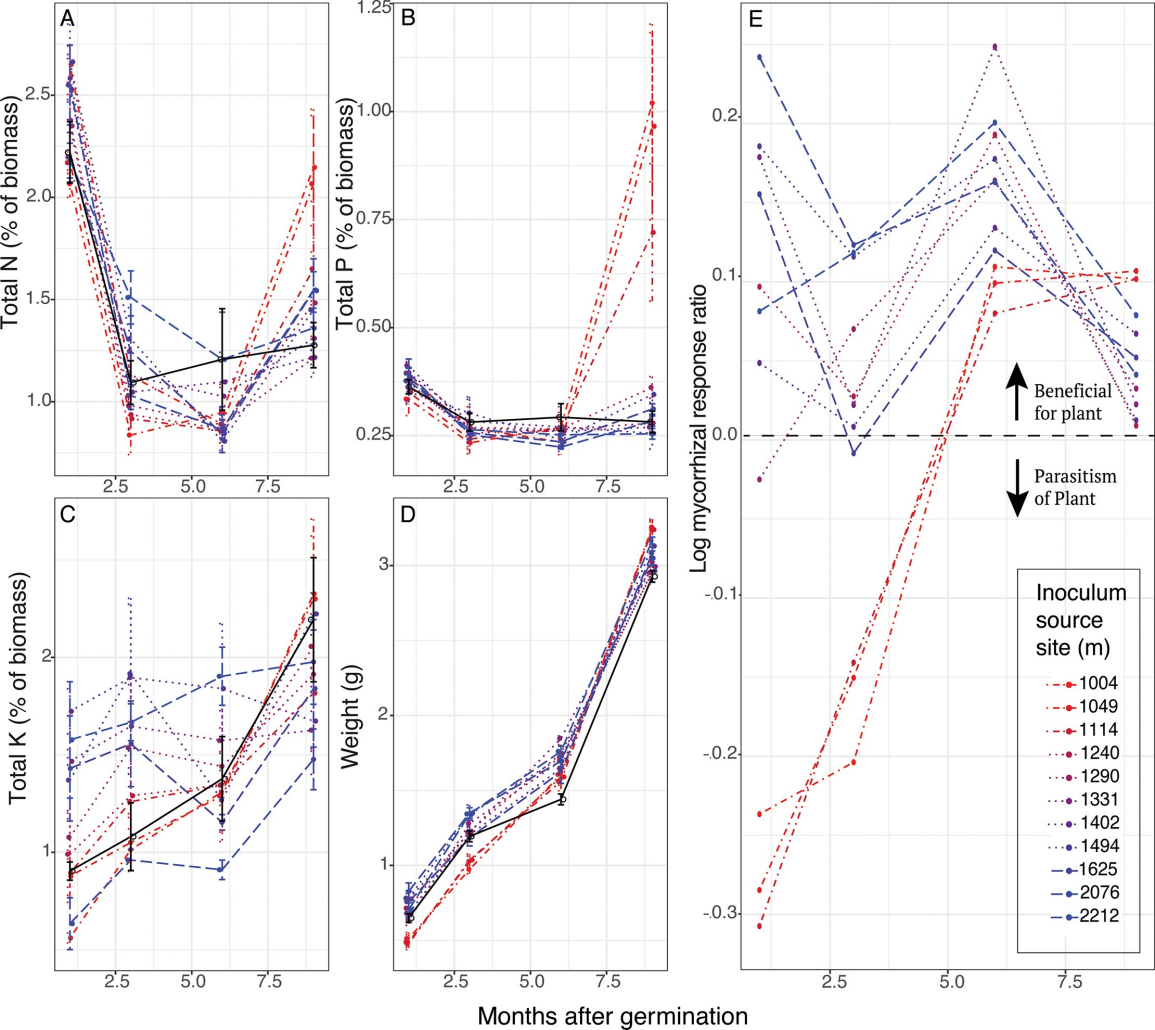
Responses of Joshua tree seedlings to treatment with different fungal communities. (A-D) Joshua tree seedlings inoculated with fungal communities from one of the 11 elevation sites, or as no-AMF control, and were destructively harvested at 1, 3, 6, and 9 months (averaged in triplicate). (E) Mycorrhizal growth response for Joshua tree seedlings (given as log mycorrhizal response ratio) at various monthly intervals following inoculation with whole fungal community inoculum from one of the eleven sites from across an elevation gradient. Log mycorrhizal response ratio is calculated as log (biomass treatment/biomass control). Colors correspond to AMF communities taken from field sites that range from warm to cool along an elevation gradient, or black for control, and points corresponding to nutrient analysis are jittered horizontally around value with error bars showing 1 SD. Line styles correspond to low-(dot-dash line), mid-(dotted line), and high-(dashed line) elevation fungal groups, or solid for the control.

The origin of fungal inoculum did not affect nutrient content of seedlings 1 month after inoculation with fungi, but plant biomass increased significantly as a function of the source elevation of the fungal inoculum (F = 4.13, df = 1, p = 2.53x10^-4^) (Fig 6) (S2 Table). Plants inoculated with fungi from all elevations showed a positive trend at three months with respect to nitrogen absorption and plant biomass respectively (F = 4.44, df = 1, p = 1.69x10^-3^; F = 5.47, df = 1, p = 5.61x10^-6^). Elevation of fungal source had no effect on nutrient absorption or plant biomass in seedlings 6 months after inoculation. The effect of the different fungal inoculum was significant for nitrogen and phosphorus levels in seedlings 9 months after inoculation with fungi (F = 7.86, df = 1, p = 0.049; F = 3.98, df = 1, p = 3.9x10^-4^). Plants inoculated with low-elevation fungi had higher average levels of nitrogen 9 months after germination, than plants grown with either the medium-or high-elevation AMF, or the control (Fig 6). This same trend was observed at 9 months for phosphorus levels, with plants inoculated with low-elevation fungi having higher levels than the mid-or, high-elevation fungi, or the control group. Potassium levels did not vary significantly with fungal treatment (F = 1.67, df = 1, p = 0.106).

The mycorrhizal growth response (MGR) of the seedlings varied with the different AMF inoculum treatments and across time (Fig 6). For the first 3 months most plants benefitted from their AMF associations, but fungi from the 3 lowest and warmest sites actually inhibited plant growth. Fungi from the low-elevation sites had a negative MGR at 1 month (-0.28 ± 0.03, n = 9), notably low when compared to the mid-elevation (0.97±0.08, n = 24) or high-elevation fungi (0.16 ± 0.08, n = 24). However, after 6 months, even the lowland AMF provided a benefit to the plants, and by 9 months those same detrimental fungi produced the greatest benefits to their hosts, with the MGR of 0.10 ± 0.002 surpassing both the mid-and high-elevation fungi (0.03 ± 0.02, and 0.05 ± 0.02, respectfully). This demonstrates that the effects of particular AMF vary according to plant developmental stage.

## Discussion

### Effect of elevation and soil properties on percent colonization

Elevation gradients represent complex variations in environmental conditions that impact fungal communities and plant-mycorrhizal interactions. We found that the percentage of Joshua tree roots colonized by AMF decreased with an increase in elevation. These results agree with findings in other systems that demonstrated AMF colonization is restricted at higher elevations [58, 59] likely due to changes in soil moisture, nutrient availability, and pH. However, percent colonization may not occur synchronously across the gradient, and future studies with multiple time points would be useful to verify the pattern that we found. Other studies have pointed to the importance of changes in plant communities along elevational gradients, shifting from species that host AMF at the lower elevations, to those that host ectomycorrhizas and ericoid mycorrhizas at higher elevations, as a major factor driving AMF colonization rates [60]. Along our 1200-m elevation gradient, Joshua trees were replaced by ectomycorrhizal pines (*Pinus monophyla*) and manzanita (*Arctostaphylos glauca*) at the higher elevation sites, which suggests a changing community of mycorrhizal types that follows plant host abundance could explain some of the observed differences. Unfortunately, we were unable to thoroughly assess colonization aggressiveness of the fungi from different elevations in greenhouse inoculated roots beyond confirming the presence of AMF in the roots among the various samples.

### Fungal community composition differs along an elevation gradient

Patterns of change in AMF community composition were associated with elevation and soil pH. Consistent with other studies, we found that AMF community distribution patterns may be shaped by variation in climate or soil resources [26, 27, 29]. Climate and soil characteristics are common predictors of fungal symbiont distribution [61] and may shape AMF distribution by affecting microbial processes such as decomposition or AMF functioning. For example, soil moisture and temperature affect AMF physiological responses such as hyphal growth rates, hyphal density, infection rates and spore propagation [62]. In controlled studies some AMF taxa proliferate under drought while others are reduced [63]. Many studies show pH can influence AMF distribution by changing soil nutrient bioavailability and metal sorption, or by directly altering the physiology of AMF [19]. AMF can also respond to changes in soil nutrients such as phosphorus concentrations, resulting in different combinations of fungal communities for a plant species exposed to different phosphorus treatments [64].

Plant communities change with elevation and can exert a strong influence on local AMF assemblages. This could be due to different plant hosts giving preferential allocation of photosynthate to the best fungal symbionts. While typically considered generalists, some AMF have been shown to be more host specific than others [65] and different AMF taxa demonstrate various plant colonization strategies [66]. Preference for different plant species could change the local fungal pool at each location along the Joshua tree elevation gradient and may contribute to some of the AMF taxa turnover that we see.

We found that AMF taxa were clustered phylogenetically by elevation, supporting a habitat filtering hypothesis. This finding supports our conceptual figure (Fig 1), namely that fungal community composition changes with the elevation gradient and in this case, resulted in a clear distinction of fungal communities for the three lowest sites, the five mid elevation sites, and the 3 highest elevation sites (Figs 3–5). It may be that changes in environmental conditions along the gradient act as filters that select for particular fungal traits. Such processes can result in phylogenetically structured communities that change with elevation [59]. Specifically, AMF from the Acaulosporaceae family were found at the highest elevation sites, which were coolest, wettest, and had the lowest pH. Fungi from this family are commonly found in lower pH environments [67] or high elevation sites [68], possibly a result of stress tolerance abilities (low pH, low nutrient soils, freezing temperatures). They sparingly use host carbon at these locations and tend towards low hyphal biomass production (both extra radical hyphae and internal root structures), instead producing diffuse hyphae [11, 66]. This result is also congruent with our findings of low root colonization by AMF at high elevations, and is consistent with phylogenetic trait conservatism within the Glomeromycota [66]. As shown elsewhere [30], Claridoglomaceae and Diversisporaceae were also more abundant at higher elevations, but less is known about the functional traits that may shape this distribution. AMF from Gigasporaceae (*Scutellospora*) were found at the lower elevation sites, which is typical for this family [67]. These fungi require substantial carbon resources because they produce large extra-radical mycelial biomass, with robust, densely aggregated hyphae [66]. The Glomeraceae were predictably distributed across all sites; this family comprises the most common AMF found in plant communities, and are the quickest and most thorough root colonizers, with the majority of fungal biomass occurring inside of the root [11, 66].

Furthermore, the principle component analysis based on OTU composition showed that the fungi at the low, mid, and high elevations clustered together in distinct groups, pointing to similarities in the AMF community composition at these locations. Our results reflect those of other studies that found fungi can experience strong habitat filtering, as evidenced by the loss or gain of taxa through taxon replacement, with increasing elevation [26–29]. As we did not analyze these fungal communities in replicate, these results are qualitative.

### Variation in plant response to mycorrhizal fungi

Joshua tree response to AMF depended on the fungal community involved as well as the developmental stage of the plant. We expected that different fungal groups would produce different symbiotic outcomes for the plant (Fig 1). Functional differences between AMF and how they colonize soil and roots can contribute to differences in host plant growth and nutrient accumulation [11]. We also found that plant growth and nutrient accumulation also changed with plant developmental time (or time following inoculation). After 9 months, plants inoculated with low-elevation AMF had higher levels of phosphorus and nitrogen then did plants inoculated with mid-or high-elevation AMF (Fig 6). Additionally, the MGR of the Joshua tree seedlings inoculated with fungi from the three lowest elevations was first negative, but after 9 months had surpassed that of plants with other fungal treatments (Fig 6). Low elevation fungi were apparently costly for the plant to initiate symbiosis, but confer benefits over time. Fungal communities from the low elevation sites were unique in that they contained fungal taxa from the Gigasporaceae and Paraglomeraceae families. AMF from Gigasporaceae quickly and extensively colonize the soil providing a greater access to soil nutrients and water [66], and a high rate of phosphorus transfer [69]. These fungi require significant carbon from the plant to build their extensive soil mycelial networks used in soil exploration and the solubilization of soil phosphorus [66]. This could explain why we see a reduction in plant mass from those treatments at earlier timepoints. An enhanced fungal network can absorb more nutrients and water leading to an increase in host photosynthetic rate [70]. This could be particularly important for the survival of desert seedlings that must establish and survive through the stressful summer months [71], especially at the hottest and driest locations. The AMF functioning may change with Joshua tree life history, acting as parasites during the rain and nutrient-abundant months after a seedling germinates, and then functioning beneficially during the hot summer months. The strong relationship between AMF community and MGR suggests that changes in the AMF community along the elevation gradient may have long term consequences for plant populations.

One inherent limitation in our study design is that this work only considers the nutrient accumulation of Joshua tree seedlings grown in greenhouse conditions that do not reflect desert climates. Greenhouse temperature was an average 17°C throughout the seedling inoculation experiment. This temperature is similar to that found at the three highest elevation sites in September, which is one of the months that Joshua tree seeds began to ripen and germinate [71]. There are multiple opportunities for seedling germination that depend on factors such as seed ripening, distribution from the indehiscent seed pods, and climate conditions [72], and are likely linked to El Niño pulse events [71]. Future work that assesses plant-AMF response under field conditions or warmer greenhouse conditions would provide useful information on how Joshua tree seedlings respond to fungal symbiosis in current and future climate scenarios. Also, the communities from the higher (more acidic soils) just may not perform as well in the cultivation substrate as fungal communities from other elevations. Another issue is that because we used whole-soil inoculum, pathogens could have been introduced that impacted plant growth. We did, however, check for AMF presence among samples in limited numbers, and no pathogenic fungal structures were noted.

### Considering future climate-induced range shifts

As Joshua tree populations shift due to the changing climate, they will encounter different fungal partners, affecting tree function and likely seedling establishment. If Joshua trees continue an upslope migration with the changing climate and lose access to lower elevation AMF communities, there could be an overall negative impact on seedling function. For example, current populations of plants with communities of Gigasporaceae and Glomeraceae at the low and mid elevations may have a higher competitive advantage. Controlled studies with other plant systems have found that these families of fungi provide complementary ecosystem functions that benefit plant hosts: Gigasporaceae provide a greater access to soil nutrients, while the extensive colonization by Glomeraceae confers reduced rates of infection by common soil pathogens [73]. Future Joshua tree shifts into high elevation areas that lack Gigasporaceae could see fewer AMF benefits to the plants realized, resulting in reduced seedling establishment and a shrinking population. AMF have limited dispersal means but fungal communities will also shift, dispersing through wind, water, and animals as the climate continues to change [12, 36, 61]. Further work testing Joshua tree-fungal combinations in the field would greatly benefit our understanding of Joshua tree symbiosis with fungal populations in future tree locations.

Assessing the outcomes of AMF and host response over both time and environmental space is helpful to make predictions about and manage conservation target species under current and future climate scenarios. Our results point to how variation across environmental gradients in mycorrhizal communities can affect the performance of host plants. However, the outcome of mycorrhizal symbiosis depends not only on the fungi, but also on the host genotypes and the environmental conditions under which the interaction takes place. An important next step would be to test elevation-specific genotypes of host plants with elevation-specific groups of fungi, tested under a range of environmental conditions, in a factorial design. Nevertheless, the low variance observed in our experiment with pooled host genotypes from across the elevation range suggests that in this particular system, host genotype may not have a major influence on the outcome. Recruitment of Joshua trees is influenced by temperature extremes [33, 36, 74–76] and drought [77]; both these factors can impact fungal community composition [78]. We have demonstrated in this study that there is a change in fungal community across an elevation gradient in JTNP and that it is associated with the change of the functional response in plants. We found that mycorrhizal impacts on Joshua trees can change from parasitic to mutualistic depending on the fungal community involved and the developmental stage of the plant. Appropriate matching of the Joshua tree fungal community with environmental conditions is an important consideration for Joshua tree restoration and assisted migration strategies.

## Supporting information

S1 Fig Sampling scheme of Joshua tree roots, soil, and fungi. (DOCX)

S2 Fig Plot of vector rank as a function of vector length used in the principal component analysis of eleven sites, based on the relative abundance of major fungal taxa. (DOCX)

S1 Table Characteristics of eleven sites along an elevation gradient in Joshua Tree National Park. These data were previously published and are presented for completeness within this manuscript.
(DOCX)

S2 Table Original data from Joshua tree fungal inoculation experiment. (XLSX)
