# Supplementary material for: Parasitism to mutualism continuum for Joshua trees inoculated with different communities of arbuscular mycorrhizal fungi from a desert elevation gradient
Source: PLoS One. 2021 Aug 27;16(8):e0256068. doi: 10.1371/journal.pone.0256068 (PMC8396742; doi:10.1371/journal.pone.0256068)
Supplement: S1 Fig — (DOCX) [file pone.0256068.s001.docx]

**S1** **Fig.** Sampling scheme of Joshua tree roots, soil, and fungi
