# Supplementary figures and images for: Parasitism to mutualism continuum for Joshua trees inoculated with different communities of arbuscular mycorrhizal fungi from a desert elevation gradient
Source: PLoS One. 2021 Aug 27;16(8):e0256068. doi: 10.1371/journal.pone.0256068 (PMC8396742; doi:10.1371/journal.pone.0256068)

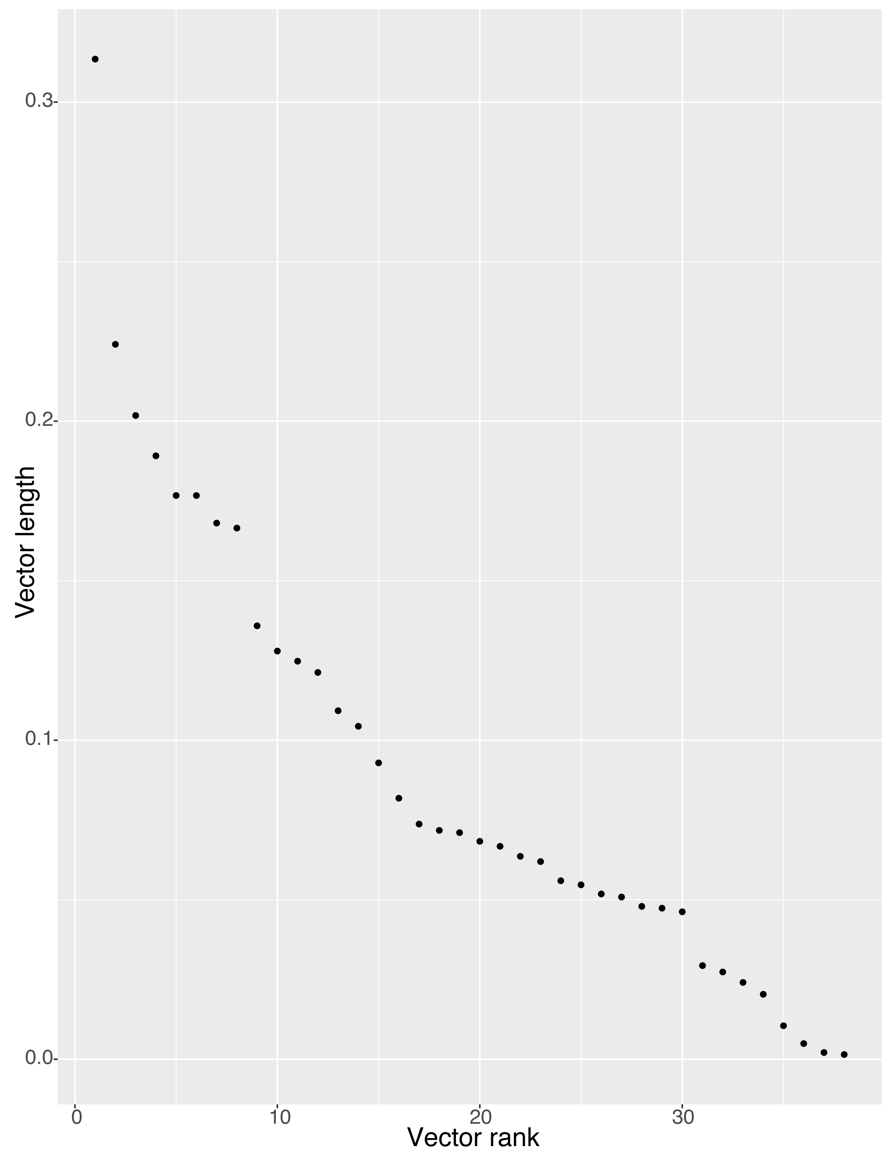


**S2** **Fig.** Plot of vector rank as a function of vector length

Supplement: S2 Fig — (DOCX) [file pone.0256068.s002.docx]
