# Supplementary material for: Parasitism to mutualism continuum for Joshua trees inoculated with different communities of arbuscular mycorrhizal fungi from a desert elevation gradient
Source: PLoS One. 2021 Aug 27;16(8):e0256068. doi: 10.1371/journal.pone.0256068 (PMC8396742; doi:10.1371/journal.pone.0256068)
Supplement: S1 Table — These data were previously published and are presented for completeness within this manuscript. (DOCX) [file pone.0256068.s003.docx]

**S1 Table. Characteristics of eleven sites along an elevation gradient in Joshua Tree National Park. These data were previously published and are presented for completeness within this manuscript.**

| Variable | Site | | | | | | | | | | |
| --- | --- | --- | --- | --- | --- | --- | --- | --- | --- | --- | --- |
|  | 1 | 2 | 3 | 4 | 5 | 6 | 7 | 8 | 9 | 10 | 11 |
| Latitude ° | 34.24 | 34.11 | 33.55 | 34.13 | 34.47 | 33.58 | 33.59 | 33.55 | 34.14 | 34.14 | 34.14 |
| Longitude ° | 116.1 | 116.0 | 116.3 | 116.1 | 116.2 | 116.1 | 116.7 | 116.1 | 116.2 | 116.4 | 116.4 |
| Elevation(m) | 1004 | 1049 | 1114 | 1240 | 1290 | 1331 | 1402 | 1494 | 1625 | 2076 | 2212 |
| Summer T (°C) | 30.2 | 29.3 | - | - | 27.3 | - | 24.4 | 23.1 | - | - | 19.9 |
| Summer RH (%) | 32.1 | 34.9 | - | - | 34.7 | - | 46.1 | 45.6 | - | - | 42.2 |
| Summer ppt (m) | 0.01 | 0.23 | - | - | 0.12 | - | 0.25 | 0.01 | - | - | 0.003 |
| Soil H_2_0 (m³/m³) | 0.01 | 0.02 | - | - | 0.05 | - | 0.11 | 0.05 | - | - | 0.14 |
| C (total) (%) | 3.03 | 0.19 | 0.2 | 0.31 | 0.21 | 0.96 | 0.34 | 0.25 | 0.29 | 0.47 | 0.52 |
| NH_4_-N (ppm) | 1.89 | 1.44 | 1.17 | 1.43 | 1.2 | 1.86 | 1.61 | 1.06 | 1.59 | 1.93 | 1.51 |
| N0_3_-N (ppm) | 5.53 | 2.03 | 3.06 | 4.26 | 2.19 | 39.1 | 2.41 | 2.19 | 4.02 | 4.42 | 1.9 |
| Olsen-P (ppm) | 10.9 | 6.3 | 8 | 9.1 | 5.4 | 19.3 | 11.4 | 7.5 | 12.9 | 22.7 | 14.9 |
| K (ppm) | 339 | 203 | 251 | 174 | 116 | 263 | 231 | 79 | 277 | 476 | 135 |
| Na (ppm) | 7 | 5 | 5 | 6 | 6 | 6 | 4 | 5 | 4 | 7 | 14 |
| Ca (meg/ 100g) | 27.2 | 3.16 | 2.63 | 3.2 | 3.03 | 7.41 | 3.75 | 4.58 | 2.85 | 3.56 | 7.47 |
| Mg (meg/ 100g) | 1.44 | 0.93 | 1.05 | 0.81 | 0.86 | 1.09 | 0.91 | 1.12 | 0.82 | 1.66 | 2.27 |
| CEC (meg/ 100g) | 29.5 | 4.62 | 4.34 | 4.49 | 4.22 | 9.2 | 5.26 | 5.93 | 4.39 | 6.47 | 10.2 |
| OM (%) | 2.87 | 0.45 | 0.42 | 0.4 | 0.4 | 1.72 | 0.58 | 0.62 | 0.81 | 1.01 | 1.28 |
| pH | 8.06 | 8.09 | 8 | 7.9 | 7.92 | 7.28 | 7.65 | 7.41 | 7.32 | 7.26 | 6.63 |

Notes: Summer T, average summer temperature; RH, relative humidity; ppt, precipitation; C, carbon; NH_4_-N, ammonium; NO_3_-N, nitrate; P, phosphorus; K, potassium; Na, sodium; Ca, calcium; Mg, magnesium; CEC, cation exchange capacity; OM, organic matter; pH, potential of hydrogen.
